# Combined Use of Vis-NIR and XRF Sensors for Tropical Soil Fertility Analysis: Assessing Different Data Fusion Approaches

**DOI:** 10.3390/s21010148

**Published:** 2020-12-29

**Authors:** Tiago Rodrigues Tavares, José Paulo Molin, S. Hamed Javadi, Hudson Wallace Pereira de Carvalho, Abdul Mounem Mouazen

**Affiliations:** 1 Laboratory of Precision Agriculture (LAP), Department of Biosystems Engineering, “Luiz de Queiroz” College of Agriculture (ESALQ), University of São Paulo (USP), Piracicaba, 13418900 São Paulo, Brazil; tiagosrt@usp.br; 2 Precision Soil and Crop Engineering Group (Precision SCoRing), Department of Environment, Faculty of Bioscience Engineering, Ghent University, Coupure Links 653, Blok B, 1st Floor, 9000 Gent, Belgium; h.javadi@ugent.be (S.H.J.); abdul.mouazen@ugent.be (A.M.M.); 3 Laboratory of Nuclear Instrumentation (LIN), Center for Nuclear Energy in Agriculture (CENA), University of São Paulo (USP), Piracicaba, 13416000 São Paulo, Brazil; hudson@cena.usp.br

**Keywords:** hybrid laboratory, soil testing, spectroanalytical techniques, precision agriculture, proximal soil sensing

## Abstract

Visible and near infrared (vis-NIR) diffuse reflectance and X-ray fluorescence (XRF) sensors are promising proximal soil sensing (PSS) tools for predicting soil key fertility attributes. This work aimed at assessing the performance of the individual and combined use of vis-NIR and XRF sensors to predict clay, organic matter (OM), cation exchange capacity (CEC), pH, base saturation (V), and extractable (ex-) nutrients (ex-P, ex-K, ex-Ca, and ex-Mg) in Brazilian tropical soils. Individual models using the data of each sensor alone were calibrated using multiple linear regressions (MLR) for the XRF data, and partial least squares (PLS) regressions for the vis-NIR data. Six data fusion approaches were evaluated and compared against individual models using relative improvement (RI). The data fusion approaches included (i) two spectra fusion approaches, which simply combined the data of both sensors in a merged dataset, followed by support vector machine (SF-SVM) and PLS (SF-PLS) regression analysis; (ii) two model averaging approaches using the Granger and Ramanathan (GR) method; and (iii) two data fusion methods based on least squares (LS) modeling. For the GR and LS approaches, two different combinations of inputs were used for MLR. The GR2 and LS2 used the prediction of individual sensors, whereas the GR3 and LS3 used the individual sensors prediction plus the SF-PLS prediction. The individual vis-NIR models showed the best results for clay and OM prediction (RPD ≥ 2.61), while the individual XRF models exhibited the best predictive models for CEC, V, ex-K, ex-Ca, and ex-Mg (RPD ≥ 2.57). For eight out of nine soil attributes studied (clay, CEC, pH, V, ex-P, ex-K, ex-Ca, and ex-Mg), the combined use of vis-NIR and XRF sensors using at least one of the six data fusion approaches improved the accuracy of the predictions (with RI ranging from 1 to 21%). In general, the LS3 model averaging approach stood out as the data fusion method with the greatest number of attributes with positive RI (six attributes; namely, clay, CEC, pH, ex-P, ex-K, and ex-Mg). Meanwhile, no single approach was capable of exploiting the synergism between sensors for all attributes of interest, suggesting that the selection of the best data fusion approach should be attribute-specific. The results presented in this work evidenced the complementarity of XRF and vis-NIR sensors to predict fertility attributes in tropical soils, and encourage further research to find a generalized method of data fusion of both sensors data.

## 1. Introduction

Proximal soil sensing (PSS) technologies allow information to be obtained on soil physicochemical attributes in a practical way without exposing chemical reagents into the environment, which is the reason why they are considered as important green tools for soil characterizations [1,2,3,4]. Studies have successfully used the information obtained using different PSS techniques operating in situ [5,6] and under laboratory conditions [7,8], suggesting practical approaches to predict and map soil attributes in agricultural fields [5,9]. Within the PSS context, particular attention has been given to the assessment of key soil fertility parameters in order to optimize the number of soil samples sent for traditional laboratory analyses [10,11].

The X-ray fluorescence (XRF) and visible and near infrared (vis-NIR) diffuse reflectance spectroscopies are promising tools for PSS applications, since both techniques allow soil analysis with minimal or no sample preparation, providing inferences about different soil constituents. There are already portable versions of these equipment that are suitable for in situ applications [12,13]. The vis-NIR diffuse reflectance spectroscopy is a widespread technique in soil science [14,15], with extensive research reporting its potential to predict mineralogical and organic attributes successfully [9,16,17,18]. Regarding soil fertility, in some cases, good results can be extended for extractable (ex-) nutrients (e.g., ex-K, ex-Ca, and ex-Mg) [7,19,20], cation exchange capacity (CEC) [19,21], base saturation (V), soil potential acidity (H + Al^3+^), and pH [19,22], which are few to mention among others. This is particularly true for secondary soil properties (spectrally inactive in the vis-NIR region), and their successful prediction is frequently attributed to correlations they have with the vis-NIR spectrally active attributes [23]. XRF elemental analysis has evolved quickly in recent years, and approaches of XRF data acquisition and processing have been developed to assess fertility attributes in agricultural soils [24,25]. XRF spectra allow for a broad characterization of soils’ elementary constitution, which has the potential to complement the information obtained with vis-NIR sensors [13,26]. The standalone use of the XRF technique has resulted in promising results for the prediction of soil texture [27,28], chemical attributes (e.g., pH, V, and CEC) [29,30,31,32], organic matter (OM) [33], and extractable nutrients (ex-K, ex-Ca, and ex-Mg) [24,34,35,36].

It is well-known that a single soil sensor alone will not promote a comprehensive characterization of all soil key fertility attributes, making it necessary to search for techniques that are complementary and suitable for work concurrently [10,37]. The combined use of different soil sensors can potentially increase the coverage of soil attributes with improved prediction accuracy compared to the single-sensor case [24,38]. Recent studies have evaluated the combined use of different PSS techniques and data fusion approaches for soil characterization [39,40,41,42]. Some studies have demonstrated that merging datasets of both XRF and vis-NIR spectroscopies can improve the quality of predictive models for soil attributes, such as total carbon (TC) and total nitrogen (TN) [43], pH, CEC, and textural attributes [44], and extractable nutrients (ex-K and ex-Ca) [26]. In addition, a recent patent of a portable apparatus that allows for the characterization of soil attributes based on a combined use of XRF and vis-NIR sensors was published [45]. Despite these recent advances, the combined use of XRF and vis-NIR sensors in the context of PSS is still at its early stages of development and further works are needed, particularly for the analysis of tropical soils that are acidic and of low fertility [11].

Although an appropriate data fusion approach is required to combine data from multiple sensors, there is still no consensus on an optimal method for predicting key soil fertility attributes. Existing techniques and frameworks of data fusion [46] include combining raw data obtained from multiple sensors [47]. Another solution is based on applying model averaging techniques using information (e.g., predictions) previously obtained by each sensor individually [48]. The model averaging approach proposed by Granger and Ramanathan (GR) [49] has been suggested for predicting soil attributes using multi-sensor data [26,48]. This method uses the individual prediction of each sensor as the input for a second calibration (e.g., using multiple linear regression (MLR)). While simple, it is as efficient as more sophisticated data fusion methods [26,50]. Another interesting data fusion approach, adapted from the literature of signal processing, is the least squares (LS) method [51]. This method considers the predictions given by soil sensors as unknown deterministic signals, since they are not random [52]. In essence, LS is equivalent to GR, provided that there is no correlation between the residuals of the sensors’ predictions. However, the residuals are correlated in practice, according to experimental results. Therefore, it is expected that LS performs at least as good as GR for cases with correlated noises. A third approach is to combine spectral data (denoted here as spectra fusion (SF)) in one matrix, which is subjected to linear or non-linear analysis. Furthermore, the majority of papers reporting the fusion of vis-NIR and XRF data focused on the prediction of one or limited number of soil attributes, e.g., soil textural attributes [44], TN and TC [43], textural attributes, pH [53], CEC [54], and chromium [48]. Although O’Rourke et al. [26] combined the vis-NIR and XRF data for the analysis of a wide range of soil attributes, they have explored the averaging data fusion methods only. To the best of our knowledge, no work exists in the literature that compares the performance of the model averaging methods with SF and LS method in soil analysis.

This work aimed at assessing the performance of the individual and combined use of XRF and vis-NIR sensors in prediction of clay, OM, CEC, pH, V, and extractable nutrients (P, K, Ca, and Mg) in tropical soils, using six different data fusion modeling approaches: (i) Combining the raw data of each sensor, followed by partial least squares (PLS) and support vector machine (SVM) regressions; (ii) applying two model averaging approaches using the GR method; and (iii) applying two least squares (LS) modeling methods. The performance of the above data fusion schemes was compared against that of the single-sensor data modeling approach.

## 2. Materials and Methods

### 2.1. Study Sites and Soil Samples

**Figure 1:**
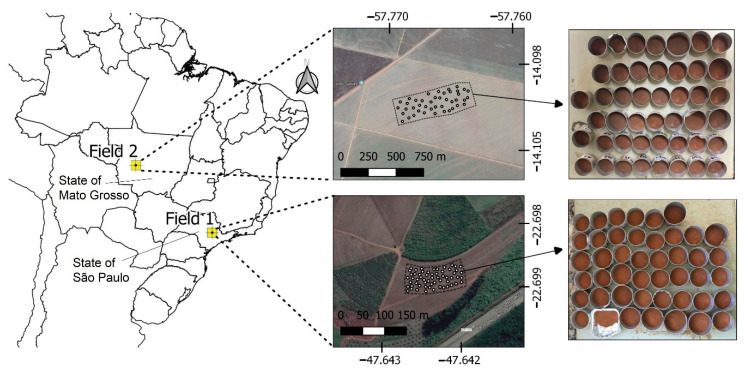
The location of the two studied agricultural fields in Brazil, and the soil samples collected from them.

A set of 102 soil samples was selected from the soil sample bank of the Precision Agriculture Laboratory (LAP) from Luiz de Queiroz College of Agriculture, University of São Paulo. The chemical analysis results of the LAP’s soil sample bank were used to select samples with wide ranges of variability of key fertility attributes in both study fields. After this selection, the samples were again subjected to laboratory chemical analyses, as described in Section 2.2, which provided the results of the reference analyses used in this work. These 102 samples were collected from two different agricultural fields from 0–20 cm depth and stored after being air-dried and sieved at 2 mm. Both fields have been under active agricultural production and have considerable textural dissimilarity. Field 1 is located in the municipality of Piracicaba, State of São Paulo, and Field 2 is situated in the municipality of Campo Novo do Parecis, State of Mato Grosso. Field 1’s soil is classified as Lixisol [55] with a clayey texture, and Field 2’s soil is classified as Ferralsol [55] with texture varying between a sandy loam and a sand clay loam. Lixisols and Ferralsols are representative and common type of soil in the Brazilian tropical areas [56]. A total of 58 and 44 soil samples were considered from Field 1 and Field 2, respectively. Figure 1 presents the location of both agricultural fields considered in this study.

### 2.2. Reference Analyses

The contents of clay, sand, OM, CEC, pH, V, ex-P, ex-K, ex-Ca, and ex-Mg were determined in a commercial laboratory of soil fertility analyses. Methods described by Van Raij et al. [57] were applied for soil analyses. Extractable nutrients (ex-P, ex-K, ex-Ca, and ex-Mg) were determined using ion exchange resin extraction. The CEC was calculated by totaling the soil potential acidity (H + Al) plus the sum of bases (ex-Ca + ex-Mg + ex-K), the former being quantified via the buffer solution method (SMP). The percent base saturation (V) was calculated by the ratio between the sum of bases and CEC. OM content was determined via oxidation with potassium dichromate solution, and pH was determined via calcium chloride solution. Clay was determined using the Bouyoucos hydrometer method in dispersing solution.

### 2.3. XRF Measurements and Selection of Emission Lines

XRF spectra were acquired using a Tracer III-SD model (Bruker AXS, Madison, EUA). This device is a portable instrument equipped with a 4 W Rh X-ray tube and a Peltier-cooled Silicon Drift Detector that has 2048 channels. The instrumental conditions suggested by Tavares et al. [24] were applied, which consisted of: (i) An X-ray tube voltage and current configured at 35 kV and 7 μA, respectively; and (ii) a scanning time (dwell time) of 90 s, performed under atmospheric pressure without filters. A cellulose pellet was used as a blank sample to ensure the contaminant-free operation of the equipment, being scanned every 30 samples.

Soil samples were scanned with the XRF sensor after being air-dried and sieved at 2 mm [35]. An XRF polyethylene cup of 31-mm diameter (Chemplex Industries Inc., Palm City, FL, USA) with the bottom sealed with a 4-μm thick polypropylene film (SPEX CertiPrep Inc., Metuchen, NJ, USA) was used to place ten grams of each sample. The samples were scanned in triplicate, moving the sample cup after each scan. The replicates were subsequently averaged for analysis.

The acquired spectra were normalized by the detector live time, after which their emission lines were evaluated in counts of photons per second (cps). Twelve emission lines (K-lines of Al, Si, K, Ca, Ti, Mn, Fe, Ni, and Cu, and the scattering peaks Rh-Lα Thomson, Rh-Kα Compton, and Rh-Kα Thomson) were selected to be used as independent variables following the criteria recommended by Tavares et al. [25], which suggested that (i) the element should be commonly found in agricultural soils; (ii) the signal-to-noise ratio (SNR) should be higher than 10; and (iii) for elements with both K and L emission lines, just their K-lines should be chosen because of the higher SNR. Finally, the nine XRF K-lines (Al-Kα, Si-Kα, K-Kα, Ca-Kα, Ti-Kα, Mn-Kα, Fe-Kα, Ni-Kα, and Cu-Kα) were normalized by the Compton peak, keeping the scattering peaks without normalization, as suggested by Tavares et al. [25].

### 2.4. Vis-NIR Measurements and Spectra Pre-Processing

The samples were scanned using a Veris vis-NIR spectrometer (Veris Technologies, Salina, KS, USA). This system uses a tungsten halogen lamp as the energy source and two spectrometers, a CCD array spectrometer (USB4000, Ocean optics, Largo, FL, USA) and an InGaAs photodiode-array spectrometer (C9914GB, Hamamatsu Photonics, Hamamatsu, Japan), to collect spectra from 343 to 2222 nm, with a spectral resolution of around 5 nm. For the vis-NIR data acquisition, the sample was placed against a circular sapphire window located in the bottom portion of a shank module. The diffused reflected energy was transmitted through a bifurcated fiber optic cable from the soil to the spectrometers. Before starting the spectra measurements, the system was calibrated using four references materials with known spectral behavior. The sensor system also self-calibrated before each spectra acquisition by collecting a dark reference measurement and a known internal reference material measurement. This self-calibration worked with a shutter system present inside the shank, which operated automatically. Further information about the equipment is provided by Christy et al. [58]. The same sample preparation applied for the XRF analysis was used for the vis-NIR measurement, which matched the standard preparation procedure adopted by the Brazilian Soil Spectral Library [22]. Each sample was scanned in triplicate, changing the position after each reading. A total of 20 spectra were recorded in each replicate, after which all the 60 vis-NIR spectra were averaged into one spectrum for further analysis.

**Figure 2:**
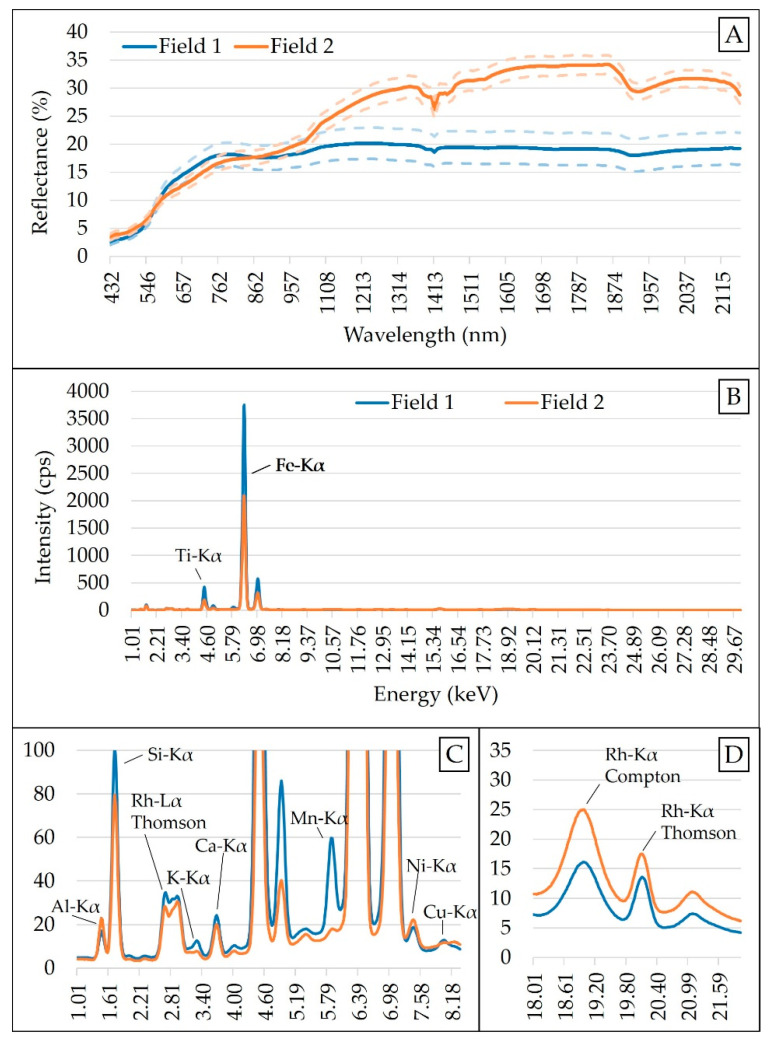
Mean spectra of Field 1 and Field 2: Obtained with visible and near infrared (vis-NIR) sensor (**A**), and X-ray fluorescence (XRF) sensor (**B**). Snapshot of the emission lines from 1.01 to 8.18 keV (**C**) and scattering peaks between 18 and 21 keV (**D**) are shown. Vis-NIR spectra are presented together with their standard deviation above and below the curve. Counts of photons per second obtained for XRF have been abbreviated as cps.

**Figure A1:**
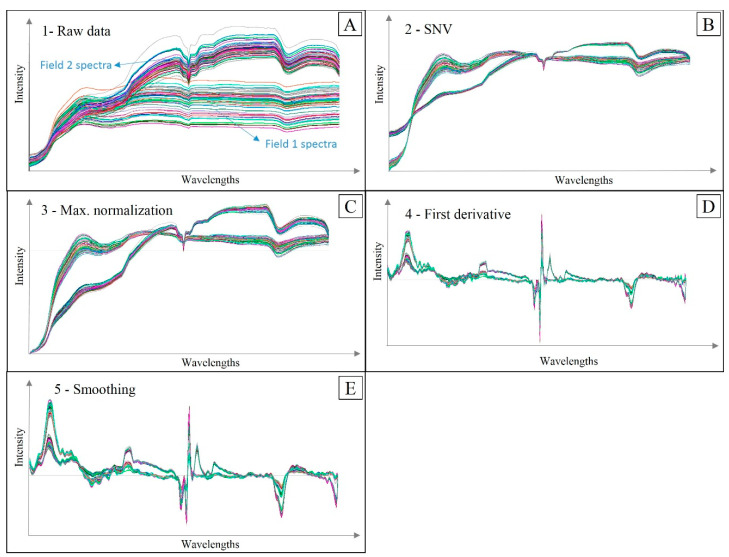
Visualization of the pre-processing sequence applied to vis-NIR spectra. Raw spectral data (**A**); spectra after standard normal variate (SNV) (**B**); spectra after SNV and maximum normalization (**C**); spectra after SNV, maximum normalization, and first derivative (**D**); spectra after SNV, maximum normalization, first derivative, and smoothing (**E**).

The raw spectra were reduced to a 437–2149 nm range due to the high presence of noise at 343–432 and 2153–2222 nm. An artifact (spectral jump) present at 1040 nm, due to the junction of the spectra obtained by the two different detectors, was corrected following the method proposed by Mouazen et al. [59]. After spectra cut and jump removal, four frequently adopted pre-processing steps were applied in the following successive order: Standard normal variate (SNV) > maximum normalization > first derivative with Savitzky–Golay (3-point window and adjusted with a second-order polynomial) > smoothing with Savitzky–Golay (3-point window and adjusted with a second-order polynomial). The standard normal variate (SNV) is a scattering correction method, which is commonly applied to vis-NIR spectra of soil samples to remove the multiplicative interferences of particle size [60]. The maximum normalization was carried out to bring all the spectra into the same numerical scale in order to create an even distribution of variances [61], while the first derivative was applied to improve the signal-to-noise ratio by highlighting weak spectral features and possible hidden information [62]. Finally, smoothing with Savitzky–Golay was applied to remove noise and improve the signal-to-noise ratio that conventional finite-difference derivatives have [61,63]. Spectra modification due to the different pre-processing steps applied in this study is shown in Figure A1, in the Appendix A. All data pre-processing steps were performed using the Unscrambler^®^ version 10.5.1 (Camo AS, Oslo, Norway). The mean XRF and vis-NIR spectra of both Field 1 and Field 2 are shown in Figure 2.

### 2.5. Modeling

The relationship between the spectra and the soil attributes was derived by predictive models using the spectral data of each sensor alone, and using the data from both sensors combined using the six fusion schemes listed above. Separate calibration equations were developed for each soil fertility attribute. The individual calibrations with each sensor alone and the calibrations with the six data fusion approaches are explained in more details in Section 2.5.1 and Section 2.5.2, respectively. All calibration and validation models were built after subdividing the dataset into two subsets with 70% (calibration set) and 30% (validation set) of data, using the Kennard–Stone algorithm [64] performed on the measured fertility attributes.

The prediction efficiency of the models was evaluated in terms of the determination coefficient (R^2^), root mean square error (RMSE), relative error (RMSE%), and the residual prediction deviation (RPD). The relative error was calculated by dividing the RMSE of each prediction by the range of the laboratory measured soil property, while the RPD was the ratio between the standard deviation (SD) of the laboratory measured soil property and the RMSE of its prediction. RPD and RMSE% allowed us to compare the predictive performance of attributes that had different units and/or scale. Four RPD classes adapted from Chang et al. [65] were used to evaluate the efficiency of the predictive models: Poor (RPD < 1.40), reasonable (1.40 ≤ RPD < 2.00), good (2.00 ≤ RPD < 3.00), and excellent (RPD ≥ 3.00) predictions. The relative improvement (RI) of the predictions achieved by the data fusion approaches was calculated (in percentage of RMSE) and compared to the best prediction obtained using a single sensor alone. This indicator shows the improvement or deterioration obtained by the joint use of the sensors, allowing its synergy for each soil attribute and data fusion approach to be assessed [26,66].

#### 2.5.1. Individual Models Using vis-NIR and XRF Sensors Alone

For the individual vis-NIR calibration model, PLS regression with leave-one-out cross-validation was used [23]. The number of latent variables adopted for each PLS model was determined for the model in cross-validation that resulted in the maximum R^2^ and lowest RMSE. For the XRF models, calibrations were built with MLR using the 12 selected emission lines as X-variables. The calibration and validation of the individual models were performed using the Unscrambler^®^ software, version 10.5.1 (Camo AS, Oslo, Norway).

#### 2.5.2. Data Fusion Approaches

Six different data fusion approaches were used to build the predictive models using both vis-NIR and XRF data combined. The SF approaches consisted of combining the spectral information of each sensor (the preprocessed vis-NIR spectra and the 12 selected XRF emission lines) followed by a regression model. Two combinations of SF were evaluated in this study, one with PLS regression and the other with SVM regression, designated as SF-PLS and SF-SVM, respectively. For the SF-PLS approach, the number of latent variables was determined according to the best cross-validation that resulted in the maximum R^2^ and lowest RMSE. The regression based on SVM is a linear machine learning method [67] that uses the most prominent data (referred to as support vectors) for regression and can be adopted for non-linear modeling by using appropriate kernels [54]. For SF-SVM, we resorted to the epsilon-SVM algorithm, which uses the radial-based kernel. This kernel includes the parameters γ ∈ [0.01, 0.1, 1, 10] and C ∈ [0.01, 0.1, 1, 10, 100] that were fine-tuned by a grid search [54].

Two calibration approaches were performed using the Granger and Ramanathan (GR) averaging method [49]. This method is simply an MLR model based on the predictions given by the individual-sensor models. In this study, two different configurations of GR were evaluated:

1. GR2, in which the predictions given by the vis-NIR and XRF individual models are fused according to the following Equation (1):
  (1) $$Y=W_{0}+\left(W_{Vis-NIR}\cdot Y_{Vis-NIR}\right)+\left(W_{XRF}\cdot Y_{XRF}\right),$$
  where *Y* is the fused (potentially more accurate) estimation of the desired soil property; *Y_vis-NIR_* and *Y_XRF_* are the corresponding predictions given by vis-NIR and XRF individual models (as described in Section 2.5.1), respectively; *W*_0_, *W_vis-NIR_*, and *W_XRF_* are the weights of the MLR determined by minimizing the mean squared error, where the first parameter is the value of the line intercept and the others are the weights of the prediction models of both sensors;
2. GR3, wherein the predictions given by the SF approach are also included in the fusion process, as described by the following Equation (2):
  (2) $$Y=W_{0}+\left(W_{Vis-NIR}\cdot Y_{Vis-NIR}\right)+\left(W_{XRF}\cdot Y_{XRF}\right)+\left(W_{SF}\cdot Y_{SF}\right),$$
  in which *Y_SF_* and *W_SF_* are, respectively, the SF predictions and their corresponding weights.

The weights in GR were calculated so that the mean squared error was minimized. To this end, normally the weights are trained using gradient descent [67,68]. Another solution is to vectorize the sensors’ predictions of the calibration set and calculate the weights by Equation (3).

(3) $${\left[W_{0},W_{Vis-NIR},W_{XRF}\right]}^{T}={\left(Y_{sensors}^{T}Y_{sensors}\right)}^{-1}Y_{sensors}^{T}$$

where $Y_{sensors}$ is the matrix including the sensors’ predictions with all of the first column elements assigned a value of one (referred to as intercept terms). It is worth mentioning that MLR does not work well when the input data are highly correlated. In this case, the weights become too sensitive to the calibration set.

Finally, two further calibration approaches were performed using LS-based fusion [52]. In LS, the correlation existing among the prediction residuals of the single sensors was computed and considered in MLR modeling. In other words, in the LS approach, the weights of the MLR models in Equations (1) and (2) were computed based on the covariance matrix of the residuals of predictions given by the individual-sensor models [52].

Similar to the GR approaches, the LS-based fusion models were examined with two combinations of inputs: (i) Using the predictions given by the vis-NIR and XRF individual models (denoted by LS2); and (ii) using the predictions of the vis-NIR and XRF individual models plus the SF model prediction (denoted by LS3). The data fusion models were calibrated and validated using Python 3.7.4. GR was evaluated by Scikit-learn package of Python [68], while the related function was developed for the evaluation of LS. This function calculated the covariance matrix based on the calibration set and used it for obtaining the regression weights.

Finally, to analyze the impact of the sensors’ errors on the data fusion accuracy, we assumed that the sensors’ errors were Gaussian and statistically independent. Then, resorting to the maximum likelihood fusion approach [52], it was straightforward to show that the fused prediction is given by Equation (4).

(4) $$y=\frac{\sigma_{2}^{2}}{\sigma_{1}^{2}+\sigma_{2}^{2}}y_{1}+\frac{\sigma_{1}^{2}}{\sigma_{1}^{2}+\sigma_{2}^{2}}y_{2},$$

where $y_{1}$ and $y_{2}$ are the predictions of sensors 1 and 2, respectively, and $\sigma_{1}^{2}$ and $\sigma_{2}^{2}$ are their corresponding variances. This indicates that the more accurate a sensor prediction is, the more it is weighted when fusing the data. On the other hand, the variance of the fused prediction is obtained by Equation (5).

(5) $$\sigma^{2}=\frac{\sigma_{1}^{2}\sigma_{2}^{2}}{\sigma_{1}^{2}+\sigma_{2}^{2}}$$

where $\sigma^{2}$ is the variance of the fused prediction and $\sigma_{1}^{2}$ and $\sigma_{2}^{2}$ are the variances of sensors 1 and 2, respectively. Equation (5) shows that the accuracy is improved by fusion since $\sigma^{2}<\min\left(\sigma_{1}^{2},\sigma_{2}^{2}\right)$. Moreover, it conveys the fact that data fusion does not necessarily improve the prediction accuracy, when one sensor performs accurately (e.g., when $\sigma_{1}^{2}=0.1\sigma_{2}^{2})$.

## 3. Results

### 3.1. Laboratory Measured Soil Properties

**Figure 3:**
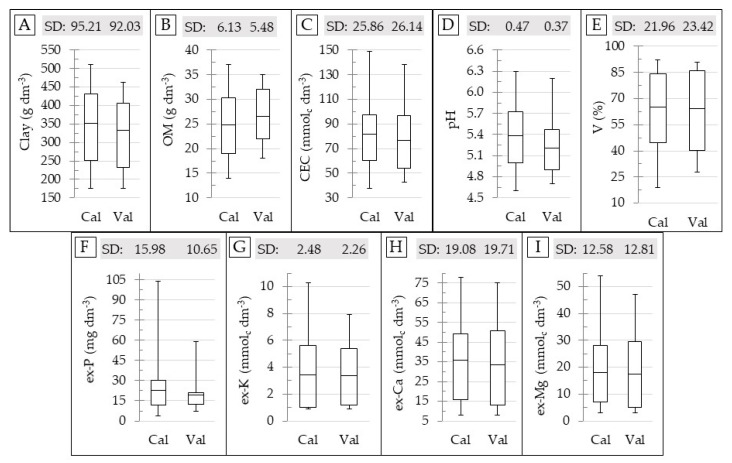
Box plot for the calibration (Cal) and validation (Val) dataset, showing the standard deviation (SD) and range of variation for the contents of clay (**A**), organic matter (OM) (**B**), cation exchange capacity (CEC) (**C**), pH (**D**), base saturation (V) (**E**), and extractable (ex-) P (**F**), K (**G**), Ca (**H**), and Mg (**I**). The skewness and kurtosis values of the datasets are presented in the Table A1, in the Appendix Section.

The boxplot and SD of the reference soil data for the calibration and validation datasets are shown in Figure 3. This figure shows that the range and SD for both calibration and validation datasets are comparable; with the only exception for the ex-P, whose SD and maximum values in the validation set are clearly lower than that in the calibration set (Figure 3F). The calibration and validation sets were selected in order to ensure a similar range and SD between them, in order to avoid negative influences on the prediction accuracy that were related to the discrepancy in characteristics of the datasets that were not related to the performance of the sensors [15,68]. Therefore, in this study, deterioration in the prediction accuracy of the ex-P was expected.

In general, the soil fertility attributes of the dataset used were not normally distributed (Table A1; Appendix Section), although modeling of the sensor’s output using sample sets with uneven distribution of soil attributes was quite common [8,69], which is known to introduce the Dunne effect with potential reduction in the prediction quality, as reported by Mouazen et al. [70].

**Figure 4:**
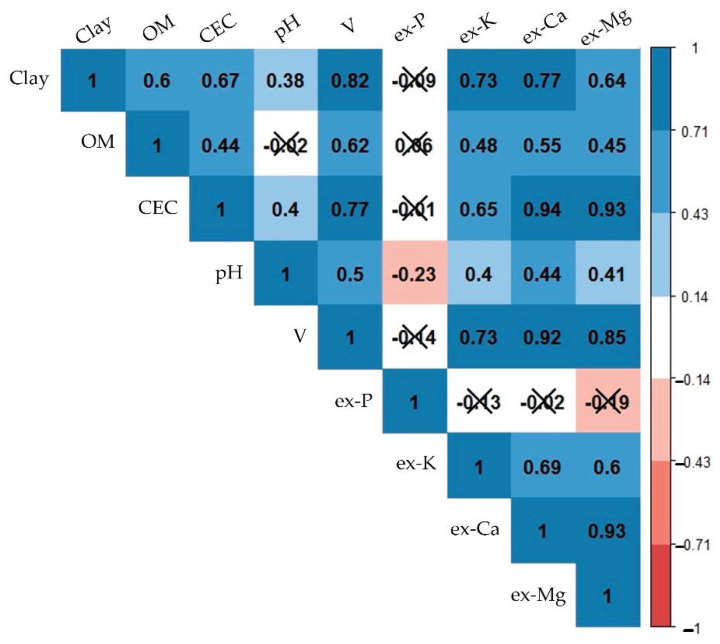
Matrix of Pearson’s correlation of the studied soil fertility attributes. Organic matter, cation exchange capacity, base saturation, and extractable nutrients were abbreviated as OM, CEC, V, and ex-P, ex-K, ex-Ca, and ex-Mg, respectively. Non-significant correlations at the probability level of 0.05 were marked with an “X”. Significant values were presented on a colour gradient, ranging from red (negative correlations) to blue (positive correlations), with the strongest correlations having the darkest colours and vice versa.

Figure 4 illustrates the relationships between the studied fertility attributes, showing the covariations among them, indicated by the Pearson’s correlation coefficient (r). Interpreting the inter-relationships between different fertility attributes aided in understanding why indirect predictions were still possible with XRF and vis-NIR data, i.e., predictions of CEC, which had no emission lines (in the case of XRF), and was a spectrally inactive component (in the case of vis-NIR). CEC, ex-Ca, and ex-Mg were closely related, with strong correlations (0.93 ≤ r ≤ 0.94). V had strong correlations with ex-Ca (r = 0.92), clay, CEC, ex-K, and ex-Mg (0.70 ≤ r < 0.90) and moderate correlations with pH and OM (0.50 ≤ r < 0.70). Ex-K possessed high correlations with clay and V (0.70 ≤ r < 0.90), moderate correlations with CEC, ex-Ca, and ex-Mg (0.50 ≤ r < 0.70), and weak correlations with OM and pH (0.30 ≤ r < 0.50). Clay content showed high correlations with V, ex-K, and ex-Ca (0.70 ≤ r < 0.90), moderate correlations with OM, CEC, and ex-Mg (0.50 ≤ r < 0.70); and a weak correlation with pH (r = 0.38). OM content had weak and moderate correlations (0.44 ≤ r ≤ 0.62) with all attributes, except for ex-P and pH, with which the correlations were non-significant. In general, ex-P and pH were the attributes that presented weaker interrelationships in comparison with the other attributes, characterized with r ranging from −0.23 to 0.06 for ex-P, and from −0.23 to 0.50 for pH.

### 3.2. Prediction Performances of Single-Sensor and Data Fusion Models

**Figure 5:**
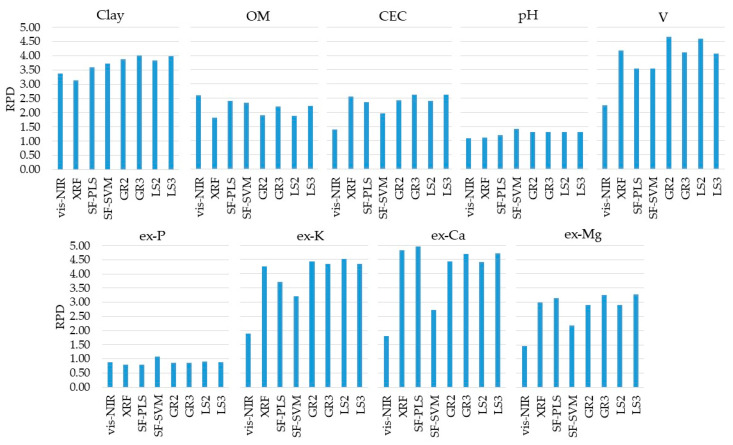
Residual prediction deviation (RPD) obtained for the predictions (using the validation set, n = 34) of clay, organic matter (OM), cation exchange capacity (CEC), pH, base saturation (V), and extractable (ex-) nutrients (ex-P, ex-K, ex-Ca, and ex-Mg) using the single visible and near infrared (vis-NIR) and X-ray florescence (XRF) data alone and combined through the six tested data fusion approaches (spectra fusion (SF-PLS and SF-SVM), Granger and Ramanathan (GR2 and GR3), and least squares (LS2 and LS3)). Detailed results of coefficient of determination (R^2^), RPD values, and root-mean-square errors (RMSE and RMSE%) are included in the Appendix Section (Table A2).

The prediction results of individual and combined vis-NIR and XRF models are presented in Figure 5 (with their details shown in Table A2, in the Appendix Section). The prediction performance of the vis-NIR sensor was satisfactory (RPD ≥ 1.40) for clay, OM, CEC, V, ex-K, ex-Ca, and ex-Mg. The best prediction performance was obtained for clay, with excellent performance (RPD = 3.37). Predictions of OM and V were of good performance, with RPD values of 2.61 and 2.26, respectively. Reasonable predictions (1.40 ≤ RPD < 1.89) were achieved for CEC, ex-K, ex-Ca, and ex-Mg, while pH and ex-P had poor prediction performances (RPD ≤ 1.10).

The same attributes satisfactorily predicted with the vis-NIR models were also satisfactorily predicted with XRF models (RPD ≥ 1.82), namely, clay, OM, CEC, V, ex-K, ex-Ca, and ex-Mg. However, CEC, V, ex-K, ex-Ca, and ex-Mg were clearly better predicted with the XRF sensor than the vis-NIR technique, with an RPD increment of 1.17, 1.92, 2.37, 3.03, and 1.54, for CEC, V, ex-K, ex-Ca, and ex-Mg, respectively (Table A2). However, clay and OM predictions were slightly better with the vis-NIR technique, with RPD values of 3.13 and 1.82, for XRF predictions, and 3.37 and 2.61, for vis-NIR predictions, respectively. Overall, the XRF sensor showed excellent performance (RPD ≥ 3.13) for the prediction of clay, V, ex-K, and ex-Mg, good performance (2.57 ≤ RPD < 2.99) for CEC and ex-Mg, a reasonable performance (RPD = 1.82) for OM, and a poor performance (RPD ≤ 1.11) for pH and ex-P.

Table 1 lists the RI (in percentage of RMSE) of the predictions calibrated using the six studied data fusion approaches (SF-PLS, SF-SVM, GR2, GR3, LS2, and LS3). Those results showed that the combined use of vis-NIR and XRF techniques by means of the different data fusion approaches tested allowed incremented improvement in predictive performance for clay, CEC, pH, V, ex-P, ex-K, ex-Ca, and ex-Mg, with a positive RI ranging from 1 to 21%. However, the data fusion did not result in improving the prediction of OM (Figure 5), with negative RI ranging between −39 and −8% (Table 1).

Comparing the predictive performance of the data fusion approaches with the best approach achieved using a single sensor alone (Table 1), clay and pH were the only attributes that showed a higher predictive performance when using all six data fusion approaches tested (SF-PLS, SF-SVM, GR2, GR3, LS2, and LS3), with the RI ranging from 6 to 16% for clay, and from 7 to 21% for pH. In turn, for CEC, V, ex-P, ex-K, ex-Ca, and ex-Mg an increase in their predictive performance was observed by using at least one of the data fusion techniques tested. In other words, not all the tested data fusion approaches have guaranteed improvement in the prediction accuracy of the studied soil fertility attributes. For example, for ex-Ca, only the SF-PLS modeling resulted in a positive RI (RI = 3%), whereas all other modeling approaches resulted in negative RI (−9% ≤ RI ≤ −2%). The same trend (but with different data fusion approaches) was observed for CEC, V, ex-P, ex-K, and ex-Mg (Table 1). It is also important to mention that regardless of the increment in prediction performance achieved with the data fusion technique used, the pH and ex-P predictions continued to be with poor performance (RPD ≤ 1.32) similar to the results from the corresponding individual sensor models.

**Figure 6:**
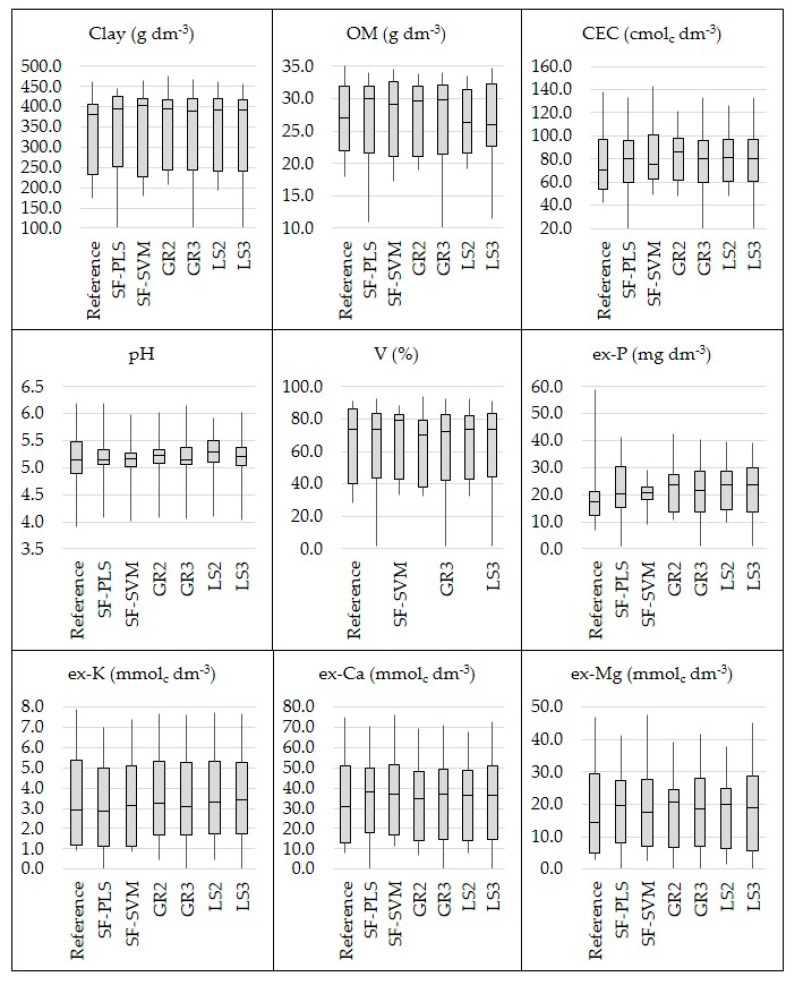
Box plot of the reference and predicted values of clay, organic matter (OM), cation exchange capacity (CEC), pH, base saturation (V), and extractable (ex-) nutrients (P, K, Ca, and Mg). The predicted values were obtained through the six tested data fusion approaches (spectra fusion (SF-PLS and SF-SVM), Granger and Ramanathan (GR2 and GR3), and least squares (LS2 and LS3)).

Comparing the six different data fusion approaches, there was no one method that stood out unanimously for all evaluated attributes (Table 1). In addition, all of them were effective in covering a similar range when comparing the dataset of predicted soil attributes with their respective reference values (Figure 6). Four out of the nine studied soil fertility attributes (clay, pH, ex-Ca, and ex-Mg) showed an increase in predictive performance with the SF-PLS approach (with RI ranging from 3 to 7%), three out of the nine (clay, pH, and ex-P) with SF-SVM (with RI ranging from 10 to 21%), four out of the nine (clay, pH, V, and ex-K) with the GR2 (with RI ranging from 4 to 16%), five out of nine (clay, CEC, pH, ex-K, ex-Mg) with the GR3 approach (with RI ranging from 2 to 16%), five out of nine attributes (clay, pH, V, ex-P, and ex-K) with the LS2 (with RI oscillating from 3 to 16%), and six out of nine attributes (clay, CEC, pH, ex-P, ex-K, and ex-Ca) with the LS3 approach (with RI oscillating from 1 to 16%). At the same time, a reduction in the predictive performance was obtained for the SF (with RI ranging from −18.40 to −8.45%) for five out of nine studied fertility attributes (OM, CEC, V, ex-P, and ex-K), for the GR2 (with RI ranging from −37.47 to −3.11%) for also five out of nine attributes (OM, CEC, ex-P, ex-Ca, and ex-Mg), for the GR3 approach (with RI ranging from −17.97 to −1.79%) for four out of nine attributes (OM, V, ex-P, and ex-Ca), for the LS2 approach (with RI ranging from −38.90 to −3.39%) for also four out of nine attributes (OM, CEC, ex-P, ex-Ca, and ex-Mg), and for the LS3 approach (with RI ranging from −17.49 to −2.15%) for three out of nine attributes (OM, V, and ex-Ca).

In general, the GR3, LS2, and LS3 data fusion approaches stood out from the others for presenting a greater number of attributes with positive RI values (e.g., positive RI for five attributes using GR3 and LS2, and six attributes using LS3). On the other hand, SF-PLS was distinguished as the best data fusion approach for the prediction of ex-Ca, with the only approach providing an increase in predictive performance in comparison with the corresponding individual XRF model (Table 1).

The weights related to the X-variables used in Granger and Ramanathan (GR2 and GR3) and least squares (LS2 and LS3) approaches are presented in Table 2. In general, the weights assigned to the XRF and vis-NIR sensors in the GR2 and LS2 methods had a similar trend to those of the individual models, in which the XRF data played a more important role for CEC, V, ex-K, ex-Ca, and ex-Mg, and the vis-NIR data for clay and OM. The ex-Ca prediction, which showed no synergy with any of the averaging model methods, was the one with the highest contrast in weight between the vis-NIR and XRF sensors (0.10 for the vis-NIR and 0.91 for the XRF in the GR2 method, and 0.08 for the vis-NIR and 0.92 for the XRF in the LS2 method). This asymmetry in weights with a higher value for the XRF corresponded to the higher accuracy of XRF compared to that of vis-NIR for the prediction of ex-Ca (as seen in Figure 5). As stated in Section 2.5.2, fusing the output of an accurate sensor with a less accurate sensor may have not improved the prediction performance. Finally, it is also worth mentioning that the predictions provided by SF-PLS played an important role in the GR3 and LS3 methods, with weights ≥ 0.34; with the only exception for the ex-P that showed weights ≤ 0.18.

## 4. Discussion

Different soil sensors can be classified based on the relationships between their data into complementary techniques, when the information provided allows the soil attributes’ coverage and/or cooperative techniques to be extended, and when the information provided presents synergism, allowing the prediction accuracy of a given soil attribute to be improved [38,46]. The discussion of the results obtained in this work was structured to address (i) the individual prediction performances obtained with the vis-NIR and XRF sensor (Section 4.1), indicating the complementarity of its information for the prediction of key soil fertility attributes; and (ii) the prediction performance improvement achieved by the data fusion approaches (Section 4.2), indicating the synergy of the sensors’ information.

### 4.1. vis-NIR and XRF Individual Performance

The XRF sensor showed a greater number of attributes predicted with good and excellent performances (i.e., with RPD ≥ 2.00). However, both vis-NIR and XRF sensors achieved satisfactory prediction performance (i.e., with RPD ≥ 1.40) for the same attributes (clay, OM, CEC, V, ex-K, ex-Ca, and ex-Mg). Using the vis-NIR sensor individually, predictions with good and excellent performances were obtained for three attributes (clay, OM, and V), while the XRF sensor alone was successful in prediction of six attributes (clay, CEC, V, ex-K, ex-Ca, and ex-Mg) with the same quality of prediction performance. Thus, by using both sensors concomitantly, through individual modeling (without using data fusion approaches), a higher number of attributes (seven attributes: clay, OM, CEC, V, ex-K, ex-Ca, and ex-Mg) was predicted with good and excellent performances compared to the individual use of each sensor alone.

The best predictive performances for OM and clay were obtained with the vis-NIR sensor, compared to those obtained with the XRF sensor. Both clay and OM are attributes with active spectral responses in the NIR region, which explains the excellent predictions obtained with the vis-NIR sensor. Clay minerals had multiple absorption features in the vis-NIR spectra (e.g., gibbsite at 2265 nm, kaolinite at 2200 and 2180 nm, and hematite and goethite at 480, 513, 650, 840, 903, and 940 nm) [71]. On the other hand, the organic compounds surface consisted mainly of carboxyl (–COOH), –OH phenolic, and alcoholic groups that are functional groups with known spectral signatures in NIR region (e.g., OH group’s features at 1414 and 1917 nm) [72]. The predictions obtained for clay and OM using the vis-NIR sensor corroborated with different studies conducted on tropical soils, which reported R^2^ values ranging from 0.75 to 0.93 for clay [17,21,73,74], and between 0.30 and 0.83 for OM [17,21,74,75]. Clay prediction in tropical soils via XRF spectroscopy is justified by the relationships that exist between the total contents of Al, Si, and Fe with kaolinite, gibbsite, and hematite clay minerals, which are minerals commonly found in Brazilian tropical soils [28]. In turn, OM predictions via XRF can be explained by its relationship with X-ray scattering peaks [33], as well as by its potential relationship with clay content [76]. Predictions with R^2^ ranging from 0.71 to 0.85 for clay [28,77], and from 0.48 to 0.98 for OM [24,28,33,34] were reported by works conducted on tropical soils using XRF sensors.

Results showed that the XRF sensor clearly outperformed the vis-NIR sensor for CEC, V, ex-K, ex-Ca, and ex-Mg prediction, although the latter also achieved satisfactory prediction performance for these attributes (Figure 5). CEC, V, ex-K, ex-Ca, and ex-Mg are considered as secondary soil attributes that have no direct spectral responses in the NIR spectroscopy range [15]. Nevertheless, vis-NIR prediction models can be successfully established for such secondary attributes, especially when they are correlated with one or more spectrally active attributes such as OM and clay [23]. In the present paper, CEC, V, ex-K, ex-Ca, and ex-Mg presented significant correlations with OM (0.44 ≤ r ≤ 0.62) and clay (0.64 ≤ r ≤ 0.82) (Figure 4), which explain the successful predictions of such secondary attributes. In Brazilian tropical soils, different studies have reported satisfactory predictions for CEC, V, ex-K, ex-Ca, and ex-Mg using vis-NIR spectroscopy sensors, with R^2^ values ranging from 0.46 to 0.92 for CEC [17,21,74], from 0.56 to 0.79 for V [74], from 0.61 to 0.94 for ex-K [73,78], from 0.42 to 0.74 for ex-Ca [7,17,74], and from 0.41 to 0.81 for ex-Mg [7,17,74].

The excellent predictions of ex-K and ex-Ca with the XRF sensor were attributed to the relationship between the total and extractable contents of these elements that existed in the evaluated soil samples (r ≥ 0.90, shown in Table A4, in the Appendix Section). It is also important to highlight that both K and Ca had clear emission lines in the XRF spectra (Figure 2C). The high correlations (r ≥ 0.92) between ex-Ca and V, and CEC and ex-Mg (Figure 4) explained the good and excellent predictions of these attributes, since they were not directly related to XRF spectra [24]. Studies on the prediction of soil fertility attributes in Brazilian tropical soils using XRF sensor reported R^2^ values ranging between 0.71 and 0.89 for ex-Ca [31,34,36,79], 0.04 and 0.81 for ex-K [31,34,36], 0.08 and 0.89 for V [31,34], 0.75 and 0.86 for CEC [24,34], and between 0.60 and 0.85 for ex-Mg [34,36].

The prediction quality of pH and ex-P was not satisfactory with any individual sensor, which can be explained by their weak association with the attributes with direct signatures in vis-NIR and XRF spectra (e.g., OM and clay, for vis-NIR spectra, and ex-K, ex-Ca, and clay, for XRF spectra). The correlations between pH with the mentioned attributes were always low (r ≤ 0.44), whereas ex-P had a non-significant correlation with all mentioned attributes (clay, OM, ex-K, and ex-Ca).

In summary, the vis-NIR sensor showed the best results for clay and OM prediction (both had direct spectral responses in NIR range), while the XRF sensor showed the best predictive models for CEC, V, ex-K, ex-Ca, and ex-Mg. Thus, the combined use of both sensors, with individual prediction models using each sensor data alone (without using data fusion), allowed us to increase the range of soil fertility attributes determined with good and excellent predictions, emphasizing the complementary relationship of their data. Finally, it is important to comment that the individual instrument models evaluated in this work were performed using traditional linear prediction models, and more sophisticated nonlinear modeling methods (e.g., machine learning and computational models) should be considered in future works, especially to deal with datasets having larger number of samples and nonlinear spectral responses.

### 4.2. Performance of Data Fusion Approches

For eight out of nine soil fertility attributes (clay, CEC, pH, V, ex-P, ex-K, ex-Ca, and ex-Mg), the combined use of vis-NIR and XRF sensors using at least one of the six tested data fusion strategies allowed achieving higher prediction performances (with RI between 1 and 21%) compared to the best individual sensor performances (Table 1). The only exception was for OM that did not show any improvement in its predictive performance using the tested data fusion strategies, which could be explained by the fact that the variance described by both sensors was not complementary for the OM characterization in our dataset. In this regard, it has been reported that data fusion will not necessarily result in optimal prediction accuracy, compared to individual sensors [26,42]. It is also interesting to highlight that although individual vis-NIR and XRF sensors for OM prediction did not show satisfactory performance (RPD ≥ 1.82), the corresponding data fusion models did not lead to any improvement in its predictive performance. On the other hand, though the prediction of pH and ex-P did not perform satisfactorily with the individual models, the models of both attributes showed a slight improvement in performance when using data fusion strategies. These results suggest that even if the vis-NIR and XRF sensors did not perform satisfactorily individually for a given attribute, their combined use could be synergistically positive for its prediction.

O’Rourke et al. [26] reported RI ranging from 15 to 44% for clay, CEC, pH, ex-K, ex-Ca, and ex-Mg after model averaging data fusion procedures (e.g., GR and variance weighted averaging), using an Australian soil dataset. Evaluating the combined use of vis-NIR and XRF techniques through different data fusion approaches, Zhang and Hartemink [53] obtained RI values of 12, 3, and 20% for clay, pH, and total carbon, respectively, for samples collected from different agricultural fields in the USA. A similar study in Chinese soils, reported an RI of 26% for CEC [54]. The synergism observed for the predictive models is explained by the contrasting design of concept of the vis-NIR and XRF sensors and spectral signatures each soil attribute may have in each sensor spectral range, which allow different variations of the soil sample constituents to be characterized, and therefore allow fertility attributes to be better inferred [38]. This feature also enables synergism for indirect characterizations, related to secondary attributes that are correlated to those with active spectral response, as seen for CEC, pH, V, and ex-Mg. It is worth noting that synergism existing in different information given by vis-NIR and XRF sensors should be exploited while taking the co-linearity and correlation among those data into account.

In general, the results obtained in this work did not identify a unique data fusion approach for exploiting the synergy between the sensors in order to predict all key fertility attributes successfully. With the exception of clay and pH, the prediction performance of data fusion models resulted in oscillated positive and negative RI values for the prediction of the same attribute. For example, the CEC prediction showed synergy with GR3 and LS3 approaches (RI = 2% for both), but its performance degraded when using GR2 (RI = −5%), LS2 (RI = −7), SF-PLS (RI = −8%), and SF-SVM (RI = −30%) models. Similarly, the V prediction accuracy improved for GR2 (RI = 10%) and LS2 (RI = 9%), while a negative RI was observed for GR3 (RI = −2%), LS3 (RI = −3%), and for both SF approaches (RI = −18% for both). The same happened for ex-P, ex-K, ex-Ca, and ex-Mg attributes. Generally speaking, the LS3 model averaging approach stood out as the best data fusion method that enabled greater number of attributes (six attributes) to be predicted with positive RI values (i.e., with better predictions, compared to the best performance obtained using a single sensor alone).

In fact, though GR and LS are conventional data fusion approaches with promising results for fusing data of vis-NIR and XRF sensors for soil analysis [26,48], they may not take advantage of all the information contained in multi-source spectra [53]. On the other hand, front-end approaches (Front-end approaches are the fusion methods which consider all available information before subjecting them to any prediction model [48,53]), such as SF, attempt to make use of all available information by merging the full data. However, this trend was not endorsed by the results of our study that showed better prediction performances for the model averaging techniques compared to the tested front-end data fusion approaches (e.g., SF-PLS and SF-SVM). In this sense, a similar behavior was also observed by O’Rourke et al. [26], who compared a front-end approach with one type of GR method. On the other hand, results by Xu et al. [48] showed comparable prediction performances between model averaging approaches (i.e., GR approach) and the outer product analysis (OPA), which is also a front-end approach for the integration of vis-NIR and XRF sensor data. Further contrasting, recent research by Zhang and Hartemink [53], which fused XRF and vis-NIR data using front-end approaches, successfully explored the synergy between sensors and achieved accurate prediction performance for soil TN and TC. The results observed in the literature clearly show that there is still no consensus on an optimal data fusion approach for vis-NIR and XRF sensor data for soil analysis. Moreover, they reinforce the hypothesis raised in this paper that the best data fusion approach might be attribute-specific.

The evaluation of the integrated use of vis-NIR and XRF sensors in tropical soils is still at its early stages of development, with the current work being the first to show a synoptic view of the potential and challenges associated with data fusion modeling for a complete characterization of key soil fertility attributes. It is undeniable that the integrated use of these sensors will be benefited if a generalized data fusion method is established, so that it can be adopted in future research. In this sense, the following suggestions are made for further research: (i) The need to assess front-end approaches, combined with more sophisticated modeling strategies, i.e., machine learning and computational models, especially for datasets with a large number of soil samples; and (ii) the need to evaluate back-end model averaging using methods that allow the estimation of the weights of each model to be optimized [79,80].

In the present scenario, one possible solution for selecting a data fusion method with optimal prediction performance is to evaluate the performance of different data fusion methods using independent subset of soil samples (independent validation). This evaluation should also include the performance of the individual models of each sensor, since data fusion models are not always the best performing when compared to individual models. The proposed strategy is similar to the one currently used for the selection of the best performing vis-NIR models for soil attribute prediction [23].

To summarize, the results obtained in this work showed the possibility of synergism between vis-NIR and XRF sensors for the prediction of the studied fertility attributes in tropical soils. The main drawback observed was that the selection of the data fusion approach should be made for each attribute, since a single approach may not allow exploring the synergism between sensors for all the attributes of interest. This finding should encourage future research to better understand the behavior of vis-NIR and XRF data fusion methods for the characterization of fertility attributes, especially in tropical soils. Finding solutions to avoid the classic disadvantage of data fusion methods related to handling large volumes of data from multiple sensors/sources should be part of the future investigations.

## 5. Conclusions

The current research assessed the performance of the individual and combined use of X-ray fluorescence (XRF) and visible and near infrared (vis-NIR) diffuse reflectance sensors to predict clay, organic matter (OM), cation exchange capacity (CEC), pH, base saturation (V), and extractable (ex-) nutrients (ex-P, ex-K, ex-Ca, and ex-Mg) in tropical soils. Six different data fusion approaches (two spectra fusion (SF), followed by partial least squares regression (SF-PLS) and support vector machine regression (SF-SVM), two Granger and Ramanathan methods (GR2 and GR3), and two methods based on least squares modeling (LS2 and LS3)) were evaluated and compared with the corresponding models obtained using data of each sensor alone. Satisfactory prediction performances (with residual prediction deviation (RPD) ≥ 1.40) using PLS regressions for vis-NIR, and multiple linear regression (MLR) for XRF were achieved for clay, OM, CEC, V, ex-K, ex-Ca, and ex-Mg using the each sensor alone. However, the combined use of both vis-NIR and XRF sensors allowed us to increase the range of soil fertility attributes that could be predicted within the good and excellent accuracy range (RPD ≥ 2.00). The vis-NIR sensor showed the best results for clay and OM prediction, while the XRF sensor showed the best prediction models for CEC, V, ex-K, ex-Ca, and ex-Mg. Among the key soil fertility attributes studied, only the pH and ex-P did not show satisfactory prediction results.

For eight out of nine soil attributes studied (clay, CEC, pH, V, ex-P, ex-K, ex-Ca, and ex-Mg), the combined use of vis-NIR and XRF sensors using at least one of the six tested data fusion approaches showed synergy, allowing for better prediction performances (with relative improvement (RI) ranging from 1 to 21%) than the corresponding predictions obtained using a single sensor data. The only exception was for OM, whose predictive performance was not improved by any of the data fusion methods tested. Our results suggest that the best data fusion approach is attribute-specific, since there was no one approach capable of exploiting the synergism between the two sensors for all attributes of interest. However, in general, the LS3 model averaging approach stood out as the best data fusion method, enabling the greatest number of attributes to be predicted with positive RI (six attributes, namely, clay, CEC, pH, ex-P, ex-K, and ex-Mg). The results presented in this work evidenced the complementarity of XRF and vis-NIR spectroscopies to predict fertility attributes in tropical soils, and encourage further research to find a generalized method of both sensors data.

## Figures and Tables

**Table 1:** Relative improvement (in percentage of root-mean-square error (RMSE)) achieved for the six studied data fusion approaches (spectra fusion (SF-PLS and SF-SVM), Granger and Ramanathan (GR2 and GR3), and least squares (LS2 and LS3)) in contrast to the best prediction obtained using a single sensor alone. The RMSE of each approach was also presented.

	Single Sensor		Multiple Sensor											
			SF-PLS		SF-SVM		GR2		GR3		LS2		LS3	
	RMSE	Techni. ^5^	RMSE	% RI ^6^	RMSE	% RI	RMSE	% RI	RMSE	% RI	RMSE	% RI	RMSE	% RI
Clay	27.32	vis-NIR	25.58	6	24.63	10	23.74	13	22.93 *	16	24.01	12	23.11	15
OM ^1^	2.10 *	vis-NIR	2.28	−8	2.34	−11	2.89	−37	2.48	−18	2.92	−39	2.47	−17
CEC ^2^	10.19	XRF	11.05	−8	13.28	−30	10.74	−5	9.99 *	2	10.9	−7	9.99 *	2
pH	0.33	XRF	0.31	7	0.26	21	0.28 *	16	0.28 *	16	0.28 *	16	0.28 *	16
V ^3^	5.6	XRF	6.63	−18	6.61	−18	5.04 *	10	5.7	−2	5.11	9	5.77	−3
ex-P ^4^	12.05	vis-NIR	13.43	−11	9.89	18	12.42	−3	12.45	−3	11.70 *	3	11.97	1
ex-K ^4^	0.53	XRF	0.61	−15	0.71	−33	0.51	4	0.52	2	0.50 *	6	0.52	2
ex-Ca ^4^	4.09	XRF	3.98 *	3	7.26	−77	4.45	−9	4.2	−3	4.46	−9	4.18	−2
ex-Mg ^4^	4.28	XRF	4.07	5	5.89	−38	4.42	−3	3.94	8	4.43	−3	3.92 *	9

^1^ Organic matter; ^2^ cation exchange capacity; ^3^ base saturation; ^4^ extractable (ex-) nutrients (ex-P, ex-K, ex-Ca, and ex-Mg); ^5^ technique; ^6^ percentage of relative improvement. The values of percentage of relative improvement (%RI) for the same soil attribute were compared and the positive RI values were presented on grayscale, with higher values having the darkest color and vice versa. RIs with negative values indicate a degradation in predictive performance, and RMSE values with an asterisk (*) indicate the approach with the lowest prediction error of all the calibrated models. The detailed results of %RI are included in the Appendix Section (Table A3).

**Table 2:** Weights assigned to XRF, vis-NIR, and SF-PLS outputs used as X-variables in Granger and Ramanathan (GR2 and GR3) and least squares (LS2 and LS3) approaches.

	GR2		GR3			LS2		LS3		
	vis-NIR	XRF	vis-NIR	XRF	SF-PLS	vis-NIR	XRF	vis-NIR	XRF	SF-PLS
Clay	0.77	0.24	0.54	0.07	0.38	0.74	0.26	0.41	0.07	0.52
OM ^1^	0.55	0.46	0.33	0.01	0.67	0.76	0.24	0.51	-0.08	0.57
CEC ^2^	0.18	0.82	0.07	0.37	0.59	0.19	0.81	0.19	0.45	0.35
pH	0.61	0.35	0.48	0.12	0.37	0.61	0.39	0.42	0.14	0.43
V ^3^	0.37	0.63	0.30	0.27	0.44	0.35	0.65	0.27	0.07	0.65
ex-P ^4^	0.46	0.62	0.42	0.48	0.15	0.55	0.45	0.50	0.32	0.18
ex-K ^4^	0.13	0.85	0.10	0.56	0.34	0.15	0.85	0.10	0.52	0.38
ex-Ca ^4^	0.10	0.91	0.08	−0.06	0.98	0.08	0.92	0.07	-0.06	1.00
ex-Mg ^4^	0.26	0.73	0.18	0.05	0.78	0.20	0.80	0.09	0.05	0.87

^1^ Organic matter; ^2^ cation exchange capacity; ^3^ base saturation; ^4^ extractable (ex-) nutrients (ex-P, ex-K, ex-Ca, and ex-Mg). The values of weights were presented on grayscale, with higher values having the darkest color and vice versa.

## Data Availability

MDPI Research Data Policies.

## Appendix A

**Table A1:** Skewness and kurtosis values for the calibration and validation dataset.

	Clay	OM ^1^	CEC ^2^	pH	V ^3^	ex-P ^4^	ex-K ^4^	ex-Ca ^4^	ex-Mg ^4^
-------------------------------------------- Calibration set (n = 68) --------------------------------------------									
Skewness	−0.22	0.14	0.46	0.50	−0.57	2.26	0.59	0.25	0.81
Kurtosis	−1.22	−1.10	−0.41	−1.02	−1.16	8.75	−0.79	−1.01	−0.14
-------------------------------------------- Validation set (n = 34) --------------------------------------------									
Skewness	−0.45	−0.11	0.53	0.83	−0.35	2.16	0.35	0.34	0.63
Kurtosis	−1.39	−1.45	−0.63	0.26	−1.62	5.78	−1.35	−1.11	−0.73

^1^ Organic matter; ^2^ cation exchange capacity; ^3^ base saturation; ^4^ extractable (ex-) nutrients (ex-P, ex-K, ex-Ca, and ex-Mg).

**Table A2:** Prediction results of the validation set (n = 34) obtained using single vis-NIR and XRF data alone and using multiple-sensor data, combined through the six tested data fusion approaches (spectra fusion (SF-PLS and SF-SVM), Granger and Ramanathan (GR2 and GR3), least squares (LS2 and LS3)).

	Clay	OM ^1^	CEC ^2^	pH	V ^3^	ex-P ^4^	ex-K ^4^	ex-Ca ^4^	ex-Mg ^4^
	-------------------------------------- R^2^ --------------------------------------								
vis-NIR	0.93	0.86	0.51	0.19	0.80	0.07	0.74	0.68	0.52
XRF	0.92	0.74	0.88	0.34	0.95	0.01	0.95	0.96	0.89
SF-PLS	0.92	0.83	0.82	0.31	0.92	0.00	0.93	0.96	0.90
SF-SVM	0.95	0.85	0.79	0.49	0.92	0.14	0.90	0.88	0.81
GR2	0.93	0.72	0.83	0.41	0.95	0.00	0.95	0.95	0.88
GR3	0.94	0.79	0.85	0.43	0.94	0.00	0.95	0.95	0.91
LS2	0.94	0.80	0.85	0.44	0.94	0.00	0.95	0.96	0.91
LS3	0.93	0.72	0.83	0.42	0.95	0.00	0.95	0.95	0.88
	-------------------------------------- RMSE --------------------------------------								
vis-NIR	27.32	2.10	18.66	0.34	10.38	12.05	1.20	10.98	8.85
XRF	29.40	3.01	10.19	0.33	5.60	13.27	0.53	4.09	4.28
SF-PLS	25.58	2.28	11.05	0.31	6.63	13.43	0.61	3.98	4.07
SF-SVM	24.63	2.34	13.28	0.26	6.61	9.89	0.71	7.26	5.89
GR2	23.74	2.89	10.74	0.28	5.04	12.42	0.51	4.45	4.42
GR3	22.93	2.48	9.99	0.28	5.70	12.45	0.52	4.20	3.94
LS2	23.11	2.47	9.99	0.28	5.77	11.97	0.52	4.18	3.92
LS3	24.01	2.92	10.90	0.28	5.11	11.70	0.50	4.46	4.43
	-------------------------------------- RMSE% --------------------------------------								
vis-NIR	9.49	12.37	19.45	22.42	16.48	23.16	17.10	16.39	20.12
XRF	10.21	17.73	10.62	22.15	8.89	25.51	7.60	6.11	9.74
SF-PLS	8.88	13.41	11.52	20.67	10.52	25.83	8.71	5.94	9.25
SF-SVM	8.55	13.77	13.85	17.41	10.50	19.02	10.11	10.84	13.39
GR2	8.24	17.00	11.20	18.67	8.00	23.88	7.29	6.64	10.05
GR3	7.96	14.59	10.42	18.67	9.05	23.94	7.43	6.27	8.95
LS2	8.02	14.53	10.42	18.67	9.16	23.02	7.43	6.24	8.91
LS3	8.34	17.18	11.37	18.67	8.11	22.50	7.14	6.66	10.07
	-------------------------------------- RPD --------------------------------------								
vis-NIR	3.37	2.61	1.40	1.10	2.26	0.88	1.89	1.79	1.45
XRF	3.13	1.82	2.57	1.11	4.18	0.80	4.26	4.82	2.99
SF-PLS	3.60	2.40	2.37	1.19	3.53	0.79	3.71	4.95	3.15
SF-SVM	3.74	2.34	1.97	1.41	3.54	1.08	3.20	2.71	2.17
GR2	3.88	1.90	2.43	1.32	4.65	0.86	4.44	4.43	2.90
GR3	4.01	2.21	2.62	1.32	4.11	0.86	4.35	4.69	3.25
LS2	3.98	2.22	2.62	1.32	4.06	0.89	4.35	4.72	3.27
LS3	3.83	1.88	2.40	1.32	4.58	0.91	4.53	4.42	2.89

^1^ Organic matter; ^2^ cation exchange capacity; ^3^ base saturation; ^4^ extractable (ex-) nutrients (ex-P, ex-K, ex-Ca, and ex-Mg). The coefficient of determination (R^2^) and residual prediction deviation (RPD) values are presented on grayscale, highlighting the highest values. The root-mean-square error (RMSE) are given in g dm^−3^ for clay and OM; in mmol_c_ dm^−3^ for CEC, ex-K, ex-Ca, and ex-Mg; in % for V; and, for ex-P, the RMSE was given in mg dm^−3^. The partial least squares (PLS) regressions of the individual vis-NIR modeling used two latent variables (LVs) for ex-K prediction, three LVs for clay, OM, CEC, V, and ex-Ca predictions, and four LVs for pH, ex-P, and ex-Mg predictions.

**Table A3:** Relative improvement (in percentage of root-mean-square error (RMSE)) achieved for the six studied data fusion approaches (spectra fusion (SF-PLS and SF-SVM), Granger and Ramanathan (GR2 and GR3), least squares (LS2 and LS3)) in contrast to the predictions obtained using the individual models of both vis-NIR and XRF sensors. The RMSE of each approach was also presented.

	Single Sensor		Multiple Sensor											
			SF-PLS		SF-SVM		GR2		GR3		LS2		LS3	
	RMSE	Techni. ^5^	RMSE	% RI ^6^	RMSE	% RI	RMSE	% RI	RMSE	% RI	RMSE	% RI	RMSE	% RI
Clay	27.32	vis-NIR	25.58	6	24.63	10	23.74	13	22.93 *	16	24.01	12	23.11	15
	29.40	XRF		13		16		19		22		18		21
OM ^1^	2.10 *	vis-NIR	2.28	−8	2.34	−11	2.89	−37	2.48	−18	2.92	−39	2.47	−17
	3.01	XRF		24		22		4		18		3		18
CEC ^2^	18.66	vis-NIR	11.05	41	13.28	29	10.74	42	9.99 *	46	10.90	42	9.99	46
	10.19	XRF		−8		−30		−5		2		−7		2
pH	0.34	vis-NIR	0.31	8	0.26 *	22	0.28	17	0.28	17	0.28	17	0.28	17
	0.33	XRF		7		21		16		16		16		16
V ^3^	10.38	vis-NIR	6.63	36	6.61	36	5.04 *	51	5.70	45	5.11	51	5.77	44
	5.60	XRF		−18		−18		10		−2		9		−3
ex-P ^4^	12.05	vis-NIR	13.43	−11	9.89	18	12.42	−3	12.45	−3	11.70 *	3	11.97	1
	13.27	XRF		−1		25		6		6		12		10
ex-K ^4^	1.20	vis-NIR	0.61	49	0.71	41	0.51	57	0.52	57	0.50 *	58	0.52	57
	0.53	XRF		−15		−33		4		2		6		2
ex-Ca ^4^	10.98	vis-NIR	3.98 *	64	7.26	34	4.45	59	4.20	62	4.46	59	4.18	62
	4.09	XRF		3		−78		−9		−3		−9		−2
ex-Mg ^4^	8.85	vis-NIR	4.07	54	5.89	33	4.42	50	3.94	55	4.43	50	3.92 *	56
	4.28	XRF		5		−38		−3		8		−3		9

^1^ Organic matter; ^2^ cation exchange capacity; ^3^ base saturation; ^4^ extractable (ex-) nutrients (ex-P, ex-K, ex-Ca, and ex-Mg); ^5^ technique; ^6^ percentage of relative improvement. The values of the percentage of relative improvement (%RI) for the same soil attribute were compared and the positive RI values were presented on grayscale, with higher values having the darkest color and vice versa. RI with negative values indicate a degradation in predictive performance, and RMSE values with an asterisk (*) indicate the approach with the lowest prediction error of all the calibrated models.

**Table A4:** Correlations between the studied soil fertility attributes and pseudo total content (ptc) of K and Ca.

	Clay	OM ^1^	CEC ^2^	pH	V ^3^	ex-P ^4^	ex-K ^4^	ex-Ca ^4^	ex-Mg ^4^
K ptc	**0.81**	**0.67**	**0.58**	**0.30**	**0.80**	−0.13	**0.90**	**0.70**	**0.58**
Ca ptc	**0.70**	**0.44**	**0.85**	**0.51**	**0.85**	0.01	**0.53**	**0.91**	**0.84**

^1^ Organic matter; ^2^ cation exchange capacity; ^3^ base saturation; and ^4^ extractable (ex-) nutrients (ex-P, ex-K, ex-Ca, and ex-Mg). Bold values indicate a significant correlation at the probability level of 0.05. Pearson’s correlation coefficient values that were significant are presented on grayscale, highlighting the highest values. Pseudo total content of K and Ca were determined following the United States Environmental Protection Agency (USEPA) Method 3051A [81], which uses a chemical dissolution of pulverized soil samples using HNO_3_ and HCl.
